# Quantitative Measurements in 3-Dimensional Datasets of Mouse Lymph Nodes Resolve Organ-Wide Functional Dependencies

**DOI:** 10.1155/2012/128431

**Published:** 2012-09-16

**Authors:** Jürgen Mayer, Jim Swoger, Aleksandra J. Ozga, Jens V. Stein, James Sharpe

**Affiliations:** ^1^EMBL/CRG Systems Biology Research Unit, Centre for Genomic Regulation (CRG) and UPF, Dr. Aiguader 88, 08003 Barcelona, Spain; ^2^Theodor Kocher Institut, Universität Bern, Freiestrasse 1, 3012 Bern, Switzerland

## Abstract

Deep tissue imaging has become state of the art in biology, but now the problem is to quantify spatial information in a global, organ-wide context. Although access to the raw data is no longer a limitation, the computational tools to extract biologically useful information out of these large data sets is still catching up. In many cases, to understand the mechanism behind a biological process, where molecules or cells interact with each other, it is mandatory to know their mutual positions. We illustrate this principle here with the immune system. Although the general functions of lymph nodes as immune sentinels are well described, many cellular and molecular details governing the interactions of lymphocytes and dendritic cells remain unclear to date and prevent an in-depth mechanistic understanding of the immune system. We imaged *ex vivo* lymph nodes isolated from both wild-type and transgenic mice lacking key factors for dendritic cell positioning and used software written in MATLAB to determine the spatial distances between the dendritic cells and the internal high endothelial vascular network. This allowed us to quantify the spatial localization of the dendritic cells in the lymph node, which is a critical parameter determining the effectiveness of an adaptive immune response.

## 1. Introduction

In recent years, 3D optical imaging techniques have become popular (confocal microscopy, two-photon microscopy, SPIM, OPT, and multifocel microscopy) [1–4]. Most of them have become standard imaging techniques. There has also been a concurrent trend towards high-resolution imaging (SIM, STED, PALM, and STORM) [5–8]. These are all capable of producing huge amounts of data in form of 3D data sets. But as the questions asked and the data sets generated to address them are often very diverse, there are few general interpretation tools available. There are a number of standard image processing programs, both commercial (Imaris, Volocity, Huygens, etc.) [9–11], freeware (MevisLab, Drishti, etc.) [12, 13], as well as open source (Fiji, ImageJ, ICY, BioImageXD, etc.) [14–17]; however, they are mostly designed for visualization and some common analysis. Often the tools or algorithms required to analyze a given data set are not available off-the-shelf and must be implemented in-house by the user. Quantification and spatial localization in 3D data sets can tell us about interactions of the structures imaged, which can be essential for deducing the mechanisms underlying features visible in the images. Pure data visualization in itself is often extremely informative, but quantification and statistical analysis of the data can indicate features or trends that are not readily apparent by visual inspection. 

During adaptive immune responses, specialized cells located in the dermis called dendritic cells (DCs), use lymphatic vessels to transport pathogen-derived material to draining peripheral lymph nodes (PLNs), where they activate T lymphocytes located close to the center of lymphoid tissue (the T-cell zone or paracortex). DC migration from skin to PLN requires expression of the promigratory receptor CCR7, which responds to secreted protein ligands CCL19 and CCL21 expressed both in lymphatic vessels as well as in the T-cell zone [18]. During their journey, dermal DCs need to cross the endothelial layer forming the lymphatic vessel lumen in the dermis. After a largely passive transport to the most proximal draining PLN, DCs cross a second barrier in the outer rim of PLN, the subcapsular sinus (SCS) [19]. CCR7 is thought to be involved in both the entry of dermal DCs into lymphatic vessels as well as their transmigration through the SCS into the deep T-cell zone. Experimentally, this process can be recapitulated by subcutaneous injection of *in vitro* activated DCs, which then migrate within the following 12–36 hours towards the nearest draining PLNs. Although such experiments uncovered an absolute requirement for CCR7 during this process, the precise contribution of CCR7 and its ligands for either of the two processes—lymphatic entry versus SCS egress—has thus far not been thoroughly investigated. 

We use selective plane illumination microscopy (SPIM) to generate 3D voxel data sets of fluorescently labeled DCs and high endothelial venules (HEV) in mouse lymph nodes. To analyze this data, which has sufficient resolution to resolve single DCs, we have implemented an algorithm in MATLAB to quantify the spatial distributions of DCs and HEV within the intact lymph node. We have used this algorithm to compare the spatial distributions between wildtype mice and the plt/plt mutant, which lacks the ligands for the CCR7 receptor. This allows us to quantify not only the number of DCs in each PLN—a measure of their ability to migrate from the dermis to the lymph node—but also their distribution within the organ, which allows us to determine how effective the DCs are at finding their way to the T-cell zone.

## 2. Materials and Methods

To achieve high-resolution imaging deep within intact organs (millimeter-sized objects) or tissues [20], SPIM is the most convenient imaging technique [2]. This technique is based on illuminating fluorescent markers in the following way: a laser light sheet penetrates the whole sample, illuminating one plane in the imaged tissue. The emission signal of the fluorophores is detected orthogonal to the incoming light sheet. As only fluorophores located in the plane of the light sheet are excited, we attain optical sectioning. Scanning the sample through the light sheet results in a 3D voxel data stack. An important prerequisite for this kind of imaging is to have a transparent sample; as is the case for the lymph nodes investigated in this study, if the tissue is opaque in its native state, it has to be cleared chemically, enforcing *ex vivo* imaging. For more detailed information about SPIM, see [2]. Labeling distinct features in the sample with different fluorescent markers generates 3D datasets with multiple channels. We applied the SPIM technology to locate and quantify leukocyte subpopulations and vascular networks in intact murine lymphoid tissue in order to examine the usefulness of our imaging approach to a biologically relevant issue. In particular, we tested the relevance of CCR7 ligands in lymphatic vessels and in the deep T-cell area for efficient DC accumulation. 

To quantify DC accumulation and their relation to the HEV, the distances to the center of the PLN and the distance to the closest neighboring HEV are calculated. The nearest neighbor calculation necessary for finding the closest HEV is done by creating and comparing voxel lists, lists of the coordinates of the centers of mass (CoMs) of the segmented DCs, and of all the segmented voxels within the HEV. After segmenting and filtering for intensity and size, for every object *p* of CH1 (DCs) the coordinates of its CoM is compared to the coordinates of all signal-containing voxels *i* of CH2 (HEV), and their respective distances are calculated as ($d_{p,i}=\sqrt{{\left(x_{p}-x_{i}\right)}^{2}+{\left(y_{p}-y_{i}\right)}^{2}+{\left(z_{p}-z_{i}\right)}^{2}}$ is the Euclidian distance between the CoM of object *p* (CoM_p_) and voxel *i* of CH2). The minimum distance of every object *p* to a CH2 voxel, min⁡_*i*_⁡(*d*_*p*,*i*_), is then used to construct the histogram. Note, that the method presented is fast only for sparse datasets (which applies here) but is not the most efficient regarding nearest neighbor search (NNS) in general (e.g., an Octree approach would be faster in denser cases). For details, see Section 3. The calculation of the distance to the center of the lymph node works similarly: we assume the center of the PLN to be the CoM of the HEV (CoM_HEV_) and then calculate dist (CoM_p_, CoM_HEV_) for every object *p*. CH2 effectively becomes a one-element voxel list, that one element being CoM_HEV_. 

Generation of dendritic cells from bone marrow (BMDCs) and PLN sample preparation was done as follows: DCs were generated by culturing bone marrow cells from tibias and femurs of C57BL/6 mice for 7-8 days in RPMI 1640/10% fetal calf serum (FCS) supplemented with FLT3 ligand as DC maturation agent. During the final 24 hours of culture BMDCs were stimulated with lipopolysaccharides (LPS) (1 *μ*g/mL; Sigma). Activated BMDCs were harvested from the plates by vigorous pipetting and washing with phosphate-buffered saline (PBS) w/o Ca^2+^ and Mg^2+^. Activation and phenotype of BMDCs were confirmed by flow cytometry staining with the DC markers CD11c (HL3), CD11b (M1/70), and CD86 (GL1) mAbs. Harvested BMDCs in RPMI1640/10% FCS were labeled with CellTracker Orange (CMTMR; 5 *μ*M for 30 min), washed and injected subcutaneously (1 × 10^6^ in 20 *μ*L RPMI 1640/10% FCS) into the hind footpads of sex-matched recipient C57BL/6 mice or C57BL/6^plt/plt^ mice. One day after BMDCs transfer, 10–15 *μ*g of Alexa594-coupled MECA-79 mAb was injected intravenously into recipient mice in order to visualize the HEV network in lymph nodes. Twenty minutes later, mice were sacrificed, the footpad-draining popliteal PLNs were excised and fixed in 0.4% PFA/PBS over night. Fixed PLNs were transferred to ice-cold PBS and cleaned carefully from the surrounding fat tissue under a stereomicroscope. Cleaned and fixed PLNs were stored in 0.1% sodium azide/PBS at 4°C before dehydration in methanol and clearing in BABB as described [21].

## 3. Results and Discussion

**Figure 1:**
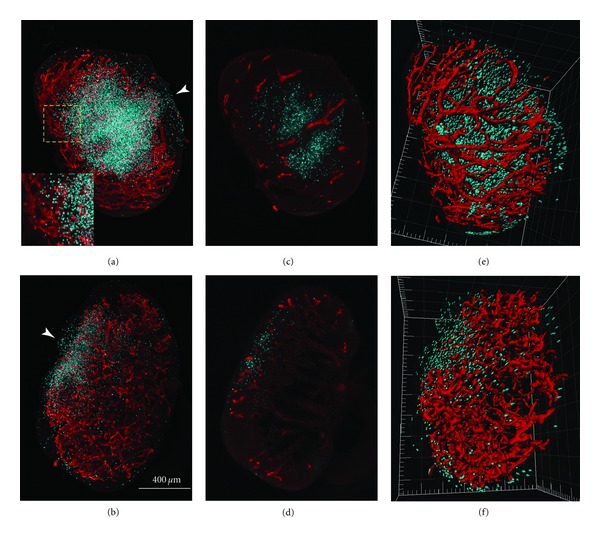
Images of PLNs from wildtype (a), (c), and (e) and plt/plt mutant mice (b) and (d), (f). HEV, red; DCs, green. (a), (b) Maximum-value projections through the voxel data sets. The inset in (a) shows a higher magnification of the region indicated. White arrowheads show the approximate positions of the afferent lymphatic vessels through which the DCs enter the PLNs. (c) and (d) Representative optical slices through the central regions of the lymph nodes shown in (a) and (b). (e) and (f); surface renderings of the data in (a) and (b).

We imaged entire draining PLNs isolated from mice that had received preactivated and fluorescently labeled DCs 18 h before the mice were sacrificed (our first channel: CH1). In addition, we imaged the PLN-specific extensive postcapillary network of HEVs as an anatomical landmark (our second channel: CH2). As seen in Figure 1, SPIM reconstructions clearly identified single DCs, which were mostly located towards the center of wildtype mouse PLNs. We were also able to detect a streak of DCs from the central accumulation to a location near the edge of the PLN, where the SCS is located. This streak most likely reflects the path that central DCs have taken after their arrival from the SCS towards the central T-cell zone. 

**Figure 2:**
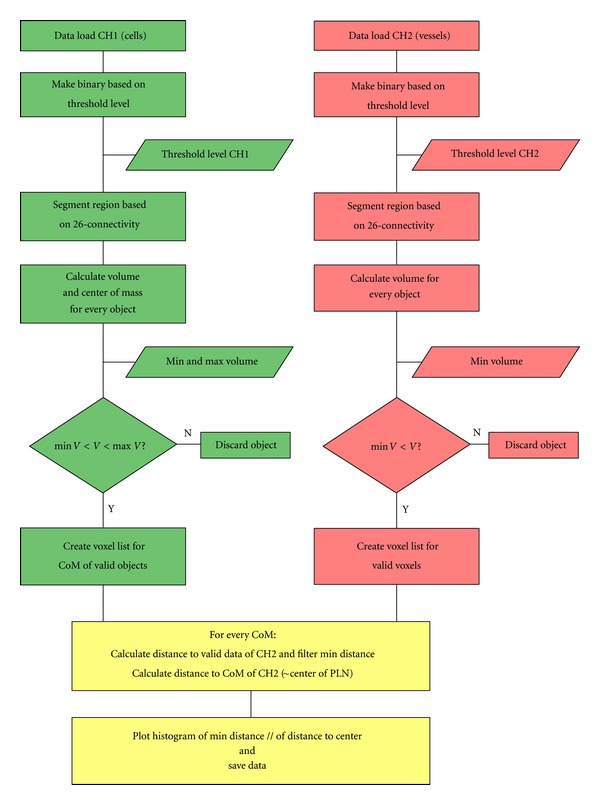
Flow diagram of the quantification algorithm. In the left path (green) dendritic cells are processed, in the right path (red), the vasculature data. The actual nearest neighbor search is done in the unified path (yellow). Abbreviations: center of mass (CoM), volume (*V*), peripheral lymph node (PLN).

Manual inspection of the single slices gives an idea of the relative distribution of the channels in this particular slice, but it is difficult to capture the 3D context (Figures 1(c) and 1(d)). A maximum value projection creates an image that shows more of the sample, but it is impossible to tell anything about the third dimension (Figures 1(a) and 1(b)). We developed an algorithm to quantify the distance of adoptively transferred DCs to the nearest point in the HEV network, as well as to the center of the PLN as a surrogate marker for accumulation in the deep T-cell area. To process the data, the algorithm pipeline (Figure 2) uses standard image processing methods, like 26-connectivity for segmentation of objects and CoM or Volume (*V*) calculations for the segmented objects. Intrinsic knowledge about the object size is used to discard signal which survived the intensity thresholding process. The choice of the threshold level influences the accuracy and precision of CoM estimation [22], but will be discussed later. In the left path of the processing pipeline (CH1, where the data of the DCs is processed), small objects either originate from pixel errors, tissue autofluorescence, or other artefacts, whereas large objects can be fluorescent impurities (e.g., dust) that have been embedded during the sample mounting procedure [23]. Both cases have to be excluded, leaving a window for objects in the size regime of DCs. We have been conservative by specifying a window of 3.2 *μ*m to 18.5 *μ*m for the DC diameter counting as a valid signal, as the average DC diameter is about 12 ± 2 *μ*m [24]. In the right path in Figure 2 (CH2, HEV), there should be ideally only one object, because it is a network of connected tubes penetrating the whole PLN. As a result, small objects can be excluded in the downstream calculations for CH2.

To explore the impact of the receptors for CCR7 on immune function within the lymph node, we took advantage of the C57BL6^plt/plt^ (plt/plt) mice, a naturally occurring mutant mouse strain, which lacks CCL19 and CCL21 protein in the T-cell zone in PLN, while maintaining expression of a second CCL21 isoform in lymphatic vessels. Although it has been previously reported that subcutaneously injected DCs accumulated with lower efficiency in the T-cell zone of plt/plt PLNs as compared to wild type PLNs [25], it remains unclear to what extent the decreased accumulation of DCs in plt/plt PLN was due to impaired entry into dermal lymphatic vessels versus decreased migration through the SCS barrier. Therefore, we performed experiments where equal numbers of DCs as used in wild type mice were injected into the footpads of plt/plt mice, followed animal sacrifice 18 h later and subsequent SPIM analysis.

**Figure 3:**
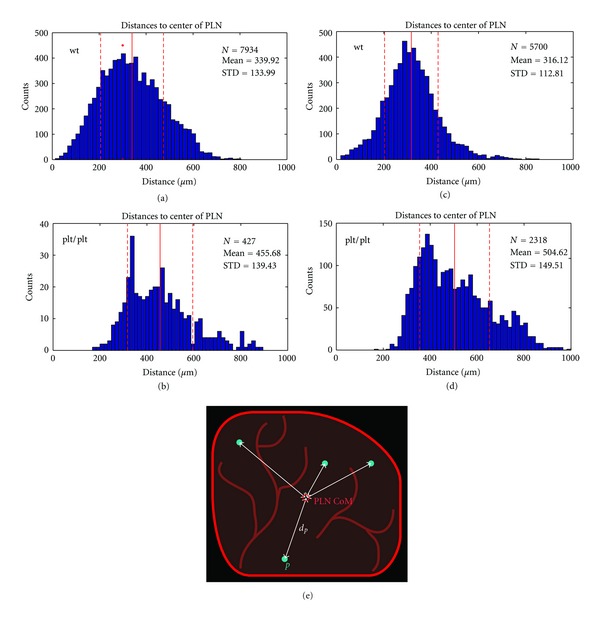
Histograms of the distances from the DCs to the center of the PLN. (a) and (c) two representative wt-PLNs, (b) and (d) plt-PLNs. The mean distance for the DCs in the wild type is smaller, indicating their location around the center of the PLN. In the plt/plt mutant, DCs remain close to their entry point in the periphery of the PLN. The red vertical line marks the mean distance, the dashed lines mark the standard deviation. (e) Schematic 2D depiction of the extent of the PLN (red) and DCs (cyan). The distances between the CoMs of the DCs and that of the PLN are indicated by white arrows.

Our whole-organ data revealed a significantly overall reduced number of DCs in plt/plt PLN, suggesting that migration of DCs into dermal lymphatic vessels was reduced in the plt/plt mice (in our case we have 5700 and 7934 identified cells for the wt/wt mice compared to 2318 and 427 cells in the plt/plt mice). More importantly, the 3D spatial distribution of DCs within plt/plt PLNs differed dramatically from wild types, with most DCs retained in the outer area in close proximity to or within the SCS. The distinct distributions for the wild type and plt/plt PLNs in the histograms in Figure 3 clearly show this effect. For the wild type (Figures 3(a) and 3(c)), the mean distance of the DCs from the center of the PLN is 330 ± 130 *μ*m, whereas for the plt/plt (Figures 3(b) and 3(d)) it is 500 ± 150 *μ*m. This result is clear despite the fact that plt/plt PLNs are generally smaller than those in wild type mice. Note that the specification of the center of the PLN is a rough estimate. It does not have to be precise in terms of specifying a real spatial center, so that first our assumption to take the HEV for calculations holds and secondly aiming for higher accuracy becomes obsolete.

To further quantify the difference between the behaviors of DCs in wild type and mutant PLNs, we assessed the distribution the DCs have concerning their minimal distance to the next HEV, so that we have a NNS-problem in a 3D dataset. The chosen method (comparing voxel coordinates) is a tradeoff between computational cost and implementation time. It is a type of linear searching and is the simplest approach to the NNS, as it has no spatial dependencies to consider (we operate in a one-dimensional space where only distances are considered). For the PLNs used in this study, generation of the samples and their preparation and scanning were a process requiring several days, whereas the computational analysis performed with our algorithm typically required tens of minutes per sample. Thus, calculation time is not the limiting factor in throughput in our system, so a linear search algorithm is completely satisfactory. If applications arise requiring reduced computational time, a more efficient algorithm regarding computation time could easily be implemented such as the use of octrees [26], or, as we look for Euclidian distances, a method similar to the vp-tree algorithm [27, 28].

**Figure 4:**
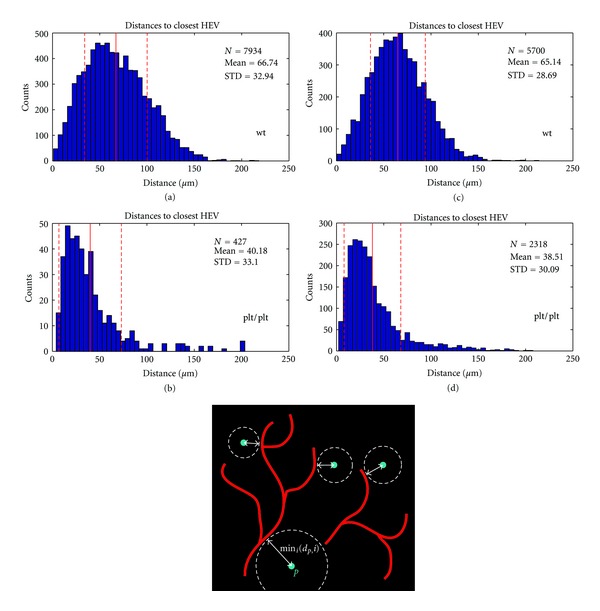
Histograms of the distances from the DCs to closest HEV, for the same PLNs as Figures 3(a) and 3(c) wt-PLN, (b) and (d) plt-PLN. Mean distance for DCs in wt-PLN is much larger than for the plt/plt mutant, reflecting the high density of the HEV in the periphery. The red vertical line marks the mean distance, the dashed lines mark the standard deviation. (e) Schematic 2D depiction of the HEV (red) and DCs (cyan). The minimum distances between the CoMs of the DCs and the HEV are indicated by white arrows and dashed circles.

In the wild type, DCs are expected to migrate from their point of entry to paracortex (or T-cell zone), where they will interact with T-cells to enable an adaptive immune response. However, this migration is dependent on DC response to the chemoattractant CCL21, which is generated in the paracortex of wildtype PLN but absent in the plt/plt mutant. We observe that the DCs in plt/plt mice, which remain located close to their entry point—the peripheral SCS—have both a smaller mean distance to the HEV (Figures 4(b) and 4(d)) and a larger distance from the center of the lymph node (Figures 3(b) and 3(d)). They do not migrate away from the peripheral region with its high HEV density (it can be observed in Figure 1(d) that the HEV is very dense in the periphery of the plt/plt PLN). With 39 ± 31 *μ*m, the plt/plt mean distance is much smaller compared to the wt/wt mean distance of 66 ± 31 *μ*m. The broader distribution in the wt/wt (compare Figures 4(a) and 4(c) to Figures 4(b) and 4(d)) is caused by DCs located both in the deep T-cell zone in the core of the PLN (where the HEV network is not as dense as in the periphery) and near the SCS (where the network is very dense).

To validate our algorithm, we digitally dissected regions of interest from the PLOs that were small enough for manual counting of the DCs by visual inspection. Comparison of the manual counts with the results of our algorithm yielded agreement to within 2-3% (data not shown). We attribute the discrepancies to statistical variations in the DC signal levels and to the difficulty in distinguishing DCs that are very close together. In any event, these small errors in DC identification are not expected to have a significant effect on the results of our analysis. The error induced by the CoM calculation to determine positions of the DCs is small as we have an almost spherical shape and a highly specific staining with a pronounced signal. The thresholding process influences the discretization bias and the averaging bias [22], but the induced error is small compared to the measured distances and does not affect our conclusions. For the same reason, we did not aim for higher precision in localization using more sophisticated localization methods.

## 4. Conclusions

In order to draw conclusions about the mechanisms behind biological behaviors of interest, it is important to quantify cell distributions comprehensively in an organ-wide context. Quantification of spatial localization can be crucial for proper understanding of the mechanisms of interactions between distinct components of an organ. We have demonstrated the application of quantitative computational analysis to an immunological question, which could be used to study 3D cell-based mechanisms in general. Fast and reliable statistics on 3D properties like the mutual distances of interacting members of distinct sets of cell types and/or morphological features in large data sets acquired in spatially extend organs or tissues can help to reveal underlying mechanisms and their functional significance. Our data quantifies the differences in the behavior of DCs in wild type and plt/plt mouse PLNs. It indicates that both the transport of DCs to PLNs (as shown by the approximately 80% reduction of DCs found in plt/plt PLNs), and their subsequent migration within these organs (as indicated by the quantification of the distribution of the DCs, in particular the approximately 50% increased distance from the center of the PLN) is compromised in the mutant mice. The approach can be applied to a wide range of biological and medical questions in context of disease mechanisms, especially considering that data from novel deep tissue imaging techniques such as SPIM are now becoming accessible. In particular, our results now make previously challenging venues of research feasible, such as the assessment of T-lymphocyte—DC interaction frequencies with physiologically low numbers of cells. 

## References

[1] Pawley JB (2006). *Handbook of Biological Confocal Microscopy*.

[2] Huisken J, Swoger J, Del Bene F, Wittbrodt J, Stelzer EHK (2004). Optical sectioning deep inside live embryos by selective plane illumination microscopy. *Science*.

[3] Sharpe J (2004). Optical projection tomography. *Annual Review of Biomedical Engineering*.

[4] Zamofing T, Hügli H Applied multifocus 3D microscopy.

[5] Shao L, Kner P, Rego EH, Gustafsson MGL (2011). Super-resolution 3D microscopy of live whole cells using structured illumination. *Nature Methods*.

[6] Klar TA, Jakobs S, Dyba M, Egner A, Hell SW (2000). Fluorescence microscopy with diffraction resolution barrier broken by stimulated emission. *Proceedings of the National Academy of Sciences of the United States of America*.

[7] Betzig E, Patterson GH, Sougrat R (2006). Imaging intracellular fluorescent proteins at nanometer resolution. *Science*.

[8] Rust MJ, Bates M, Zhuang X (2006). Sub-diffraction-limit imaging by stochastic optical reconstruction microscopy (STORM). *Nature Methods*.

[9] Bitplane http://www.bitplane.com/.

[10] PerkinElmer http://www.perkinelmer.com/pages/020/cellularimaging/products/volocity.xhtml.

[11] SVI http://www.svi.nl/HomePage.

[12] Fraunhofer http://www.mevislab.de.

[13] Limaye A http://sf.anu.edu.au/Vizlab/drishti/.

[14] http://fiji.sc/wiki/index.php/Fiji.

[15] Rasband W http://rsbweb.nih.gov/ij/.

[16] http://icy.bioimageanalysis.org/.

[17] http://www.bioimagexd.net/.

[18] Von Andrian UH, Mempel TR (2003). Homing and cellular traffic in lymph nodes. *Nature Reviews Immunology*.

[19] Forster R, Braun A, Worbs T (2012). Lymph node homing of T cells and dendritic cells via afferent lymphatics. *Trends in Immunology*.

[20] Helmchen F, Denk W (2005). Deep tissue two-photon microscopy. *Nature Methods*.

[21] Sharpe J, Ahlgren U, Perry P (2002). Optical projection tomography as a tool for 3D microscopy and gene expression studies. *Science*.

[22] Van Assen HC, Egmont-Petersen M, Reiber JHC (2002). Accurate object localization in gray level images using the center of gravity measure: accuracy versus precision. *IEEE Transactions on Image Processing*.

[23] Quintana L, Sharpe J (2011). Preparation of mouse embryos for optical projection tomography imaging. *Cold Spring Harbor Protocols*.

[24] Davidson DJ, Currie AJ, Reid GSD (2004). The cationic antimicrobial peptide LL-37 modulates dendritic cell differentiation and dendritic cell-induced T cell polarization. *Journal of Immunology*.

[25] Gunn MD, Kyuwa S, Tam C (1999). Mice lacking expression of secondary lymphoid organ chemokine have defects in lymphocyte homing and dendritic cell localization. *Journal of Experimental Medicine*.

[26] Szeliski R (1993). Rapid octree construction from image sequences. *CVGIP: Image Understanding*.

[27] Xu H, Agrafiotis DK (2003). Nearest neighbor search in general metric spaces using a tree data structure with a simple heuristic. *Journal of Chemical Information and Computer Sciences*.

[28] Kumar N, Zhang L, Nayar SK What is a good nearest neighbors algorithm for finding similar patches in images?.

